# Gingival crevicular fluid as a periodontal diagnostic indicator- II: Inflammatory mediators, host-response modifiers and chair side diagnostic aids

**Published:** 2013-03-25

**Authors:** G Gupta

**Affiliations:** Reader, Department of Periodontics, Institute of Dental Studies and Technologies, Modinagar

**Keywords:** Biomarkers, Inflammatory mediators, Host response modifiers

## Abstract

There has been a steady growing trend during the last few decades to develop tools to monitor periodontitis, in the field of oral disease diagnosis. Since GCF has the chance of being closely approximated to the periodontal tissues where periodontal disease begins, it seems to provide more information than markers in saliva. In response to bacterial infection, host production of inflammatory mediators, may be the trigger for periodontal disease progression. Existing paradigms in the biology of periodontitis have supported the detection of elevated levels of these mediators in GCF. This article is the Part II of the review that deals with inflammatory mediators and host-response modifiers as the potential biomarkers present in gingival crevicular fluid (GCF) and the chair side point-of-care diagnostic aids applicable to monitor periodontal inflammation.

## Introduction

Periodontal disease is a chronic bacterial infection characterized by persistent inflammation, connective tissue breakdown and alveolar bone destruction. In addition to local periodontal tissue involvement, chronic infection of periodontium with continuous up-regulation of pro-inflammatory responses and immune mediators may contribute to systemic sequel including diabetes, pre term low birth weight babies, lung inflammation, arthritis and cardiovascular diseases. These contributing inflammatory mediators have been detected in the gingival tissues; gingival crevice fluid (GCF) of patients affected by periodontitis, and qualitative changes in the composition of these biomarkers could have a diagnostic and therapeutic significance.

**Table 1:** 

Cytokines
Interleukin -1 a
Interleukin -1β
Interleukin -1 ra
Interleukin-2
Interleukin-6
Interleukin-8
Tumor necrosis factor a
Interferon a
Prostaglandin E2
Leukotriene B4
Acute-phase proteins
Lactoferrin
Transferrin
a2 -Macroglobulin
a 1-Proteinase inhibitor
C-reactive protein
Autoantibodies
Anti-desmosomal antibody
Antibacterial antibodies
IgG1, IgG2 , IgG3 , IgG4
IgM
IgA
Plasminogen activator (PA)
PA inhibitor-2 (PAI-2)
Substance P
Vasoactive intestinal peptide
Neurokinin A
Neopterin
Platelet -Activating Factor
CDl4
Cystatins
Calgranulin A (MRP-8)

 Cytokines - Lipopolysaccharide (LPS) is a key microbial stimulus that will trigger the host response at periodontal disease sites. It is a cell-wall component of gram-negative bacteria, shed out of the biofilm in membrane vesicles. Locally, it triggers monocytes to release inflammatory mediators (Prostaglandin E2, Thromboxane B, Interleukins -1, -6 and -8, Tumor necrosis factor) that increase the local destruction of the connective tissues structural elements. Therefore, levels of monocytic inflammatory mediators (including prostaglandin E2, interleukin-1, and tumor necrosis factor) in GCF may well represent the ideal markers of disease activity at a site level [**1-6**].

 Interleukin-1 (IL-1) is a potent bone-resorbing cytokine formerly known as the osteoclast-activating factor. Interleukin-1 is primarily produced by activated macrophages or lymphocytes but it may also be released by other cells, including mast cells, fibroblasts, keratinocytes, endothelial cells and its production is stimulated by bacterial lipopolysaccharide [**7**]. It is found in two active forms IL-1α and IL-1β. Once secreted, IL-1 may activate lymphocytes, incite macrophage chemotaxis and prostaglandin production, and stimulate osteoclastic resorption of bone [**8**]. IL-1 has been detected in both periodontal tissues and GCF in patients with periodontal disease [**9**]. Interleukin-6 is an inflammatory cytokine that leads to bone remodeling [**10**]. Tumor necrosis factor - α is produced by activated macrophages in response to bacterial LPS. It has similar effects on osteoclast as IL-1 but is less potent. Both IL-1 and TNF-α induce production of proteinases in mesenchymal cells, including MMPs, which contribute to connective tissue destruction [**11**].

 IL-1, IL-6, and TNF- α are found in significant concentrations in GCF from periodontally diseased sites. Reductions in IL-1 concentrations are associated with successful treatment [**12**]. Elevated levels of IL-6 in GCF are associated with sites that do not respond well in initial nonsurgical phases of therapy [**13**]. Increasing severity of periodontitis is associated with increased concentrations of IL-1 and decreasing concentrations of IL-1ra [**14**]. Preliminary findings also suggest a possible inverse relationship between TNF-α [**4**] and IFN-γ [**5**] and a positive relationship between IL-6 [**6**] and tissue inflammation, however appropriate longitudinal studies relating their presence and concentration in GCF to active periodontitis have yet to be conducted.

 IL-8 was originally described as a chemotactic protein isolated from stimulated human blood mononuclear cells. This cytokine is induced and secreted from many different cells, including monocytes, lymphocyte, fibroblasts, endothelial cells, epithelial cells and synovial cells. IL-8 is a potentially important mediator regulating PMN activity in the crevicular environment. This cytokine induces shape change, chemotaxis, a rise in intracellular free calcium, the respiratory burst, and exocytosis of primary and secondary granules from these cells. In addition, IL-8 can induce adhesion of PMN to endothelial cells, transendothelial migration of these cells as well as up-regulation of complement receptors 1 and 3 (CR1 and CR3) on the surface of human PMN [**15**]. Decreased IL-8 concentrations at diseased sites may reflect the reduced anti-bacterial host defense activity at that site [**16**].

 Interferon α - It is thought to promote anti-bacterial IgG activity. Since IL-1 may promote Th 1 activity through increased IL-2 production, IL-1 may also increase IFN- α production. Also, since IFN- α is produced by Th 1 cells and Th 1 cells are responsible for cell-mediated immune responses. A decrease in the effective GCF concentrations of IFN- α at diseased sites may signify a lowered cell-mediated immune response, which may result in decreased specific anti-periodontopathic bacterial activity [**17**]. 

 lL-2 has primarily been associated with an autocrine factor for T cells,[**18**] although recent data indicate the ability of this factor to stimulate B lymphocytes, a report by Pilon et al. [**19**] provided evidence for IL-2 in GCF, suggesting activation of T lymphocytes in the periodontium.

 RANTES - It is a member of a superfamily of proinflammatory cytokines, activates monocytes, eosinophil and basophilic leukocytes, [**20**] inducing chemotaxis and the release of other cell mediators [**21**]. This chemokine is involved in the development of the gingival inflammatory response by mediating the recruitment and activation of leukocytes. RANTES is present in GCF of patients with periodontitis and is undetectable in healthy subjects [**22**].

 Prostaglandin E2 - Prostaglandins are synthesized by most mammalian cells and have been implicated as components of the inflammatory reaction, being able to produce vasodilatation, bone resorption and inhibition of collagen synthesis. The primary cells responsible for PGE2 production in the periodontium are macrophages and fibroblasts. It is elevated in GCF from periodontally diseased sites demonstrating inflammation and attachment loss [**23**]. 

 Leukotriene B4 (LTB4) - It is a membrane derived lipid mediator formed from arachidonic acid via the 5-lipoxygenase enzymatic pathway, possesses a variety of biological actions during inflammatory response [**24**]. It is produced from a variety of cells including neutrophils, macrophages, and eosinophils. LTB4 plays a unique functional role by its effects on neutrophils. This potent lipid mediator can activate neutrophil chemotaxis, aggregation, and adhesion to endothelial cells; lysosomal enzyme release; and superoxide production. It increases vascular permeability, [**24**] and can modulate lymphocyte functions by stimulating INF-γ and IL-2 production from T cells, and increase IL-1 synthesis by monocytes. All of these LTB4-dependent mechanisms may contribute to the inflammation and destruction of the bone and connective tissue in periodontal disease. Increased LTB4 levels in GCF and periodontal tissues samples of periodontally diseased sites suggest that this lipid mediator is important in regulating inflammatory responses in the human periodontal tissues [**25**].

## Acute-phase proteins

 Lactoferrin (Lf)- is an iron-binding multifunctional glycoprotein present in most biological secretions and in neutrophils. One of the earliest and very well documented functions of Lf is the antibacterial effect against representative periodontopathic bacteria, Aggregatibacter actinomycetemcomitans, Porphyromonas gingivalis, and Prevotella intermedia [**26**]. The mechanism of the antibacterial activity of Lf is complex and several lines of evidence indicate that beside iron chelating, it involves a direct action on bacteria and/or the activation of the immune system. T sai et al. [**27**] found that GCF lactoferrin levels in chronic periodontitis patients were higher than in periodontally healthy individuals.

 Transferrin- TF is a serum derived iron binding glycoprotein whose main function in humans is the transport of iron between sites of absorption, storage, utilization and excretion [**28**]. In GCF, it might function as an antibacterial agent by producing an iron-limiting environment and its levels are significantly increased during diseased state [**29**].

 C-reactive protein (CRP) - Although produced by hepatocytes, there is evidence that CRP is present on cells associated with acute connective tissue inflammation [**30**]. Rapid and dramatic rise in blood levels of CRP occurs following trauma or disease producing inflammation. Its physiological role is to bind to phosphocholine expressed on the surface of dead or dying cells (and some types of bacteria) in order to activate the complement system via the C1Q complex [**31**]. The rapid rise of CRP in serum occurs following exposure to interleukin-1 [**30**], which is a potent bone resorber, also found in GCF. High levels of CRP are associated with chronic and aggressive periodontal diseases. 

 Antidesmosomal antibodies - During periodontal disease, the cells of junctional epithelium attempt to establish and maintain cell-cell contact. To promote contact, the desmosomes, specialized electron dense plaques, cement extracellular regions of cell membranes together. Interactions between desmosomal proteins are important in the formation of the desmosomes and the maintenance of stable cell adhesion [**32**]. Adhesion between epithelial cells may be destabilized during inflammation. Alteration in cytokeratin expression in inflamed gingiva may affect the formation of desmosomes, making the desmosomes more sensitive to inflammation-mediated proteolysis or reactive with autoantibodies. In periodontal disease, autoantibodies may be produced by antigen-specific and polyclonal activation of lymphocytes [**33**]. If they are expressed in periodontal disease, autoantibodies against desmosomal components may inhibit the assembly of desmosomes in the junctional epithelium. New cell-cell contact would be inhibited and apical migration of the epithelial attachment may be promoted. The presence of elevated titers of anti-desmosomal antibodies in GCF appears to distinguish periodontitis from unaffected sites [**34**].

Antibacterial antibodies - GCF accumulates serum immunoglobulins from the dense plasma cell infiltrate present during the established stages of periodontitis. As many patients with periodontitis, especially those with the aggressive forms, produce antibodies to infecting periodontopathic bacteria, many attempts have been made to find an association between the presence of antibodies in GCF and periodontitis.

 IgG antibodies have been considered important in preventing periodontal destruction in patients with aggressive and chronic periodontitis. IgG is composed of four distinct subclasses. The IgG subclasses differ not only in their distribution in normal serum, but also in their biologic properties and the nature of the antigens, which elicit their production [**35**]. One important biologic function of IgG involves the activation of the complement cascade, which can initiate a number of protective reactions including phagocyte chemotaxis, membranolytic attack on susceptible gram-negative organisms, immune adherence, and opsonization. Whereas IgG1 and IgG3 are potent complement activators, IgG2 is comparatively weak in this regard. IgG4 appears to be inactive in fixing complement via the classical pathway. IgG2 is the major immunoglobulin subclass that reacts with bacterial carbohydrates and lipopolysaccharides, and it may serve as a good opsonin [**36**]. An impairment of IgG2 responses may increase the risk of bacterial disease including periodontal disease. In periodontal lesions, IgG-positive plasma cells predominate, which is reflected in IgG, being the primary immunoglobulin in GCF in advanced clinical stages of periodontitis [**37**].

IgM antibodies exert anti-bacterial activities by enzyme, toxin and viral neutralization, complement-induced cytolysis, agglutination, opsonization and inhibition of attachment to host tissue. It is less numerous but are first to be released in the presence of an antigen [**38**]. Mean amount of IgM in GCF was significantly greater at baseline than at 3 months after root planning and scaling [**39**].

 IgA in GCF may have anti-inflammatory effects. It has been demonstrated to be a potent inhibitor of complement activation by IgG [**40**]. In the gingival sulcus, IgA could function by preventing adherence of pathologic microorganisms to the tooth surface and thereby helping to eliminate these bacteria from the subgingival environment. IgA may also function by binding directly to IgA specific Fc receptors on PMN, which in turn could down regulate the activity of these cells [**41**]. Concentration of IgA is elevated in patients with less severe periodontal disease. This elevated concentration of IgA suggests that it may play an important protective mechanism in the periodontium and furthermore, it may be useful in identifying patients who are protected, or at low risk for future periodontal disease [**42**].

Plasminogen activator (PA) and PA inhibitor-2 (PAI-2) - The fibrinolytic system is important in inflammatory reactions in that it regulates pericellular proteolysis. This may facilitate connective tissue breakdown and spread of inflammatory lesions. The system is activated by plasminogen activators (PA), which are serine proteases catalyzing the conversion of the inactive proenzyme plasminogen into the active enzyme plasmin. Plasmin is an aggressive enzyme, which can in turn activate procollagenases and other metalloproteases [**43**], thereby participating in the tissue destruction seen at inflammatory lesions. PAs can be produced by several types of cells, among them, macrophages [**44**]. The activity of the PAs is balanced by two similarly distinct, specifically acting plasminogen activator inhibitors (PAI-1 and PAI-2). Both PAs and PAI are found in GCF in notably high concentrations, from inflamed gingival sites compared to healthy sites [**45**]. Within the range of gingival inflammation studied, the PAs seemed to be balanced by a higher concentration of PAI-2, since no change was seen in the molar ratio of PAs to PAI-2 [**46**].

Substance P and Neurokinin A - Neuropeptides are biologically active peptides generated primarily in neurons. Substance P (SP) and neurokinin A (NKA) are members of the tachykinin family of neuropeptides. SP and NKA are derived from the same gene, may have very similar roles [**47**], but have distinct receptors [**48**]. SP and NKA are stored in secretory granules of sensory unmyelinated c-type fibers and significant amounts are present both in primary sensory neurones and in their peripheral branches [**49**]. Antidromic stimulation leads to the release of these peptides from the peripheral nerve endings which can lead to vasodilatation, increased microvascular permeability and plasma extravasation, resulting in neurogenic inflammation [**50**]. Experimental studies have demonstrated that the application of SP can cause neurogenic inflammation of the oral mucosa [**51**]. Immunohistochemical studies have shown that SP is localized in nerve fibers of gingival connective tissue [**52**]. The release of these peptides into the gingival connective tissue could be followed by the diffusion through the epithelium and subsequently into the gingival crevice or periodontal pocket [**53**]. 

 Vasoactive intestinal peptide (VIP) – It is a ubiquitous peptide, with a multiplicity of actions, including modulation of both natural and acquired immunity [**54**]. Although VIP affects a variety of immune functions, its primary immunomodulatory function is anti-inflammatory in nature. Two sources of VIP have been described, nerves (both central and peripheral) and leukocytes. Nerve fibers positive for VIP have been identified in gingival tissue from both normal and periodontitis-affected sites [**55**]. Periodontal treatment results in changes in the levels of the proinflammatory neuropeptides SP and NKA in GCF [**56**]. In periodontitis subjects, VIP is significantly elevated in periodontitis-affected sites compared with the levels in clinically healthy sites. Non-surgical periodontal treatment in these patients resulted in an improvement in the periodontal condition, accompanied by a reduction in the levels of VIP in periodontitis sites compared to those found in clinically healthy sites [**57**].

 Neopterin - It is an actual molecular mass compound belonging to the class of pteridines, is an early and valuable biomarker of cellular immunity. Neopterin is produced by human macrophages after the induction by IFN- γ secreted by T lymphocytes which play an important role in periodontal diseases [**58**]. Ozmeric et al. [**59**] have reported that GCF neopterin concentration is higher in periodontitis patients.

 Platelet Activating Factor (PAF) - It is a potent biologically active phospholipid mediator capable of exerting marked proinflammatory responses [**60**]. Major cell sources for the production of PAF are thrombocytes, neutrophils, macrophages, eosinophils, and epithelial cells [**60**]. It is linked to many inflammatory and immune responses including platelet stimulation, neutrophil and monocyte activation, increased vascular permeability, and smooth muscle contraction. PAF activates IL-1 production by monocytes, inhibits IL-2 synthesis from T lymphocytes, and modulates B-cell functions [**60**]. All of these PAF-dependent mechanisms may contribute to the destruction of the bone and connective tissue in periodontal disease together with other host-derived mediators. Overall, elevated PAF levels in both GCF and gingival tissue samples of specific periodontal diseases support the destructive role of this lipid mediator in human periodontal tissues [**61**].

 CD14 - It was originally described as a myeloid differentiation antigen detected on mature monocytes/macrophages and neutrophils [**62**]. It is a key molecule responsible for the innate recognition of bacteria by host cells and functions as an important LPS receptor on monocytes/macrophages and neutrophils [**63**]. CD14 exists in 2 forms, a membrane-bound form (mCD14) and a circulating soluble form (sCD14). sCD14 appears to have 2 main functions: 1) neutralizing the LPS-LBP complex, thereby preventing further stimulation of the inflammatory response, and 2) interacting with cells that do not normally express CD14, such as endothelial cells and epithelial cells, enabling LPS activation of these cells [**64**]. The release of cytokines IL-1β, TNF-α, and PGE2 and the expression of cell adhesion molecules such as E-selectin and ICAM-1 are all initiated in the response of host cells to CD14 complexed with microbial LPS [**62**]. Thus, CD14 may play an important role in the regulation of inflammatory and immunological responses, and the tissue destruction mediated by LPS in the pathogenesis of periodontitis. Increased sCD14 concentrations in GCF was found in the study by Jin and Darveau [**65**].

Cystatins- They comprise a superfamily of proteins, subdivided into 3 families [**66**]. Family 1 cystatins (cystatin A and B) are homologous 1-domain proteins of 11 kDa, which are found mainly intracellularly. Family 2 cystatins are proteins of 14 kDa and contain 2 disulfide bridges. These cystatins are found mainly in secretory fluids and are represented in saliva by acidic (S, SA), neutral (SN, D) and basic (C) cystatins [**67**]. Family 3 cystatins, the kininogens, are intravascular multifunctional proteins with 3 cystatin-like domains. Cystatins are the endogenous inhibitors that regulate the activity of cysteine proteinases. Cystatin activity in GCF is caused mainly by cystatin A [**68**].

Calgranulin A (MRP-8) - MRP-8 and MRP-14 are both expressed in circulating PMN and macrophages, but not in macrophages found in normal healthy tissue. In acute inflammatory reactions, MRP-8 is never found in the tissue, whereas in chronic inflammation, MRP-8 and MRP-14 are expressed by macrophages in the inflamed tissue [**69**]. MRP-8 is a major responsive protein involved in the pathogenesis of periodontitis, functioning as a powerful chemotaxin leading to further influx of PMN into the inflammatory focus in the gingival crevice/ pocket. Alternatively, evidence that MRP-8 is chemotactic for periodontal ligament cells [**70**] may indicate a role for MRP-8 in periodontal remodeling. MRP-8 is detectable in GCF of chronic periodontitis, without MRP-14 [**71**].

## Chairside diagnostic kits

 Molecular analysis of GCF elution can be time consuming while most of the GCF analytic assays are laboratory based and usually cannot be performed in a chair-side manner. These procedures, as well as GCF sampling, are technically demanding and the GCF volume can be very small (1-5 μl). Despite these apparent diagnostic and technical disadvantages, GCF is still considered as a candidate potential oral fluid for the development of adjunctive non-invasive chair-side point of-care diagnostic technology, especially because tissue destructive MMPs and their bioactive regulators can conveniently be measured by distinct catalytic and non-catalytic immunoassays from GCF.

**Table 2:** Commercially available chairside diagnostic kits

ASSAY	KIT	MANUFACTURER/SUPPLIER	FUNCTION
Bacterial enzymes & host enzymes	BANA periodontal test	Ora Tec Corporation Manassas (USA)	It utilizes the BANA test for bacterial trypsin like proteases
	Periocheck (ASTech) [**72**]	CollaGenex Pharmaceuticals, Newtown, PA	Detects presence of neutral proteinases i.e. Collagenase
	PerioScan	Oral B Laboratories	Detects enzymatic activity of Aggregatibacter actinomycetemcomitans, T forsythus, P gingivalis
Immunological detection	Evalusite	Kodak Eastman Company (Switzerland)	Immunological detection of antigens of Aggregatibacter actinomycetemcomitans, P intermedia, P gingivalis using antibodies (ELISA)
Biochemical Identification	Prognostic [**72**]	Dentsply	Aids in detection of serine proteinases and elastases
	Biolise [**73**]	SLT-Labinstruments, Crailsheim, Germany	Aids in detection of elastase
	Periogard [**74**]	Colgate	Detects the presence of AST
	Pocket watch	SteriOss®, San Diego, CA, USA	Detects aspartate aminotransferase through colorimetric detection
	TOPAS	Affinity Labelling Technologies (USA)	Detects toxins derived from anaerobic metabolism and measures GCF protein level

### Future directions for biomarker based diagnostics of periodontitis

 Through the biomarker discovery process, new therapeutics have been designed linking therapeutic and diagnostic approaches together, especially in the area of host modulatory drugs for periodontal disease treatment. Moreover, new diagnostic technologies, such as microarray and microfluidics, are now currently available for risk assessment and comprehensive screening of biomarkers. The future is bright for the use of rapid, easy-to-use diagnostics that will provide an enhanced patient assessment that can guide and transform customized therapies for dental patients, leading to more individualized, targeted treatments for oral health.

## Conclusion

Periodontal tissue destruction derived from chronic periodontitis occurs as consequence to the activation of immune-inflammatory response of the host to bacterial challenge. Major events comprise pro-inflammatory cytokine production and collagenolytic MMPs leading to soft periodontal tissue breakdown. These cytokines along with bone MMPs, will lead ultimately to octeoclastogenesis and alveolar bone resorption. Qualitative and/or quantitative changes in oral fluid biomarkers might be useful as adjunctive diagnostics and treatment of periodontitis.
